# Feasibility of Prehospital Teleconsultation in Acute Stroke – A Pilot Study in Clinical Routine

**DOI:** 10.1371/journal.pone.0036796

**Published:** 2012-05-18

**Authors:** Sebastian Bergrath, Arno Reich, Rolf Rossaint, Daniel Rörtgen, Joachim Gerber, Harold Fischermann, Stefan K. Beckers, Jörg C. Brokmann, Jörg B. Schulz, Claas Leber, Christina Fitzner, Max Skorning

**Affiliations:** 1 Department of Anesthesiology, University Hospital Aachen, RWTH Aachen University, Aachen, Germany; 2 Department of Neurology, University Hospital Aachen, RWTH Aachen University, Aachen, Germany; 3 Department of Intensive Care Medicine and Intermediate Care, University Hospital Aachen, RWTH Aachen University, Aachen, Germany; 4 Department of Medical Statistics, University Hospital Aachen, RWTH Aachen University, Aachen, Germany; University of Regensburg, Germany

## Abstract

**Background:**

Inter-hospital teleconsultation improves stroke care. To transfer this concept into the emergency medical service (EMS), the feasibility and effects of prehospital teleconsultation were investigated.

**Methodology/Principal Findings:**

Teleconsultation enabling audio communication, real-time video streaming, vital data and still picture transmission was conducted between an ambulance and a teleconsultation center. Pre-notification of the hospital was carried out with a 14-item stroke history checklist via e-mail-to-fax. Beside technical assessments possible influences on prehospital and initial in-hospital time intervals, prehospital diagnostic accuracy and the transfer of stroke specific data were investigated by comparing telemedically assisted prehospital care (telemedicine group) with local regular EMS care (control group). All prehospital stroke patients over a 5-month period were included during weekdays (7.30 a.m. –4.00 p.m.). In 3 of 18 missions partial dropouts of the system occurred; neurological co-evaluation via video transmission was conducted in 12 cases. The stroke checklist was transmitted in 14 cases (78%). Telemedicine group (n = 18) vs. control group (n = 47): Prehospital time intervals were comparable, but in both groups the door to brain imaging times were longer than recommended (median 59.5 vs. 57.5 min, p = 0.6447). The prehospital stroke diagnosis was confirmed in 61% vs. 67%, p = 0.8451. Medians of 14 (IQR 9) vs. 5 (IQR 2) stroke specific items were transferred in written form to the in-hospital setting, p<0.0001. In 3 of 10 vs. 5 of 27 patients with cerebral ischemia thrombolytics were administered, p = 0.655.

**Conclusions:**

Teleconsultation was feasible but technical performance and reliability have to be improved. The approach led to better stroke specific information; however, a superiority over regular EMS care was not found and in-hospital time intervals were unacceptably long in both groups. The feasibility of prehospital tele-stroke consultation has future potential to improve emergency care especially when no highly trained personnel are on-scene.

**Trial Registration:**

International Standard Randomised Controlled Trial Number Register (ISRCTN) ISRCTN83270177 83270177.

## Introduction

Stroke is a major cause of death worldwide and the third leading cause of death in the U.S. [1]. Approximately one-third of stroke survivors remain permanently disabled [1], [2]. Optimizing the “symptom to treatment” interval is essential to improving stroke treatment and outcome. Telemedicine networks between community hospitals and stroke centers provide expertise to remote sites and can enhance safety and quality of care [3]–[7]. Through telemedicine, especially real-time teleconsultation, patient outcome can be improved if no vascular neurologist is on site. Systems incorporating video consultation seem to be superior to telephone consultation alone [3], [4], [5].

Emergency medical services (EMS) are core elements in the chain of survival from acute stroke [6]. The American Heart Association recommends the implementation and evaluation of mobile telemedicine systems to support stroke care in EMS [7], [8]. Despite abundant technological opportunities, the use of telemedicine for stroke in EMS remains uncommon. Video transmission from an ambulance to an expert may improve diagnostic accuracy, appropriate triage and time to thrombolysis, if indicated. Thus far, prehospital video transmission has been realized in only one project; this group detected an improved time management in simulated stroke scenarios compared with real patients [9], [10]. The clinical value of this proceeding is still unknown [8].

With this background in mind, a mobile prehospital teleconsultation system was designed that included video transmission in real time from the ambulance to a teleconsultation center, where experienced physicians provided medical and organizational support [11]. The system was developed to enable support in all kinds of emergencies, but a specific process chain was implemented for acute stroke. In this study, the feasibility of this system and approach was researched under clinical routine conditions.

## Methods

**Figure 1:**
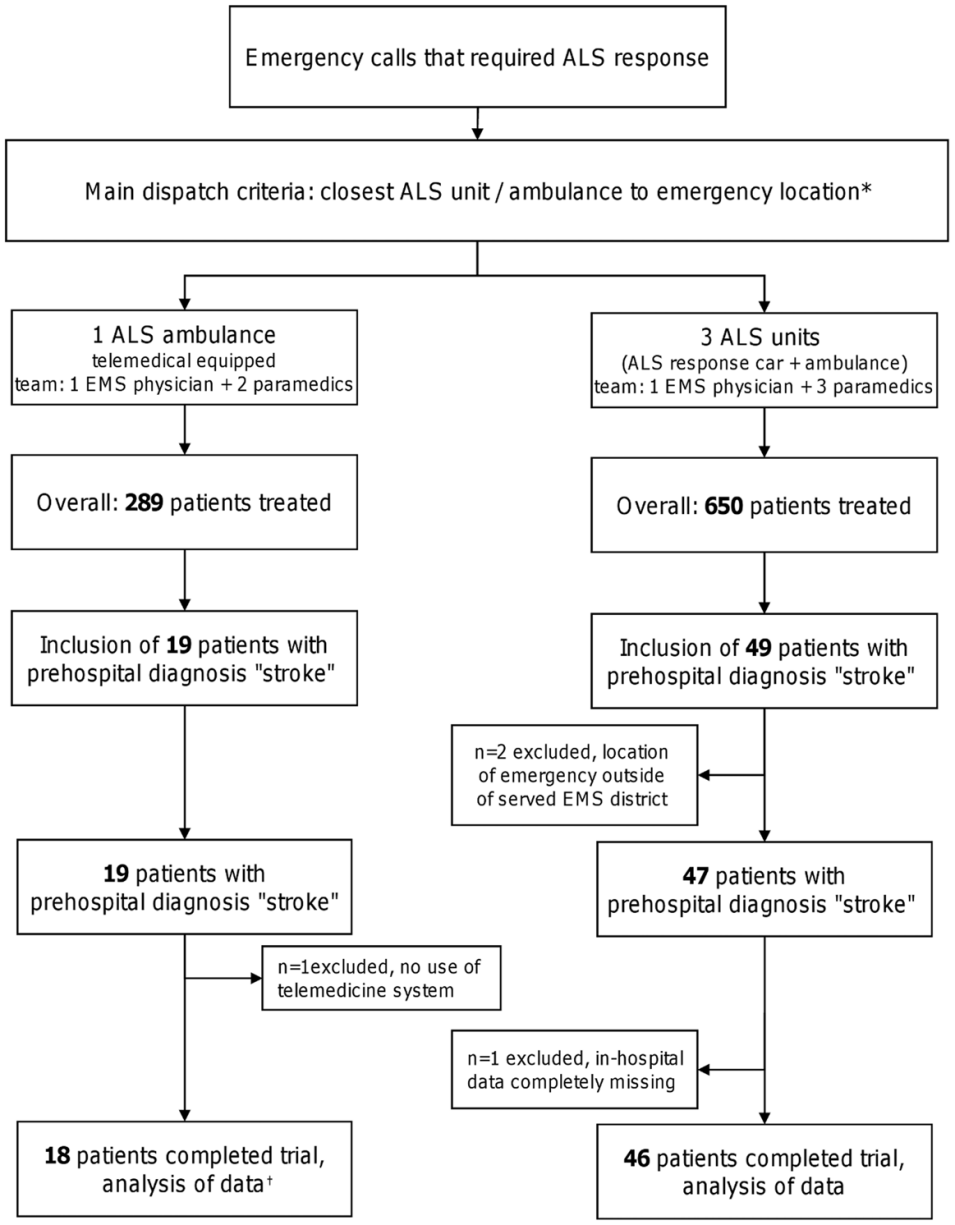
Trial flow. ALS, Advanced Life Support; EMS, Emergency Medical Service. * if telemedical and standard ambulance had the same distance to emergency location: primary dispatch of telemedical ambulance, regardless of the type of emergency † technical and organizational assessments.

This prospective study was conducted between May 3, 2010 and September 30, 2010 during weekdays (7.30 a.m. to 4.00 p.m.) in the EMS of Aachen, Germany. During this phase one advanced life support (ALS) ambulance was equipped with a teleconsultation system, and a novel prehospital process chain for acute stroke was carried out. Beside technical assessments possible influences on prehospital processes and early in-hospital processes were investigated by comparing telemedically assisted prehospital care (telemedicine group) with local regular emergency medical care (control group) under equivalent circumstances in a non-randomized setting. Additionally stroke specific in-hospital data were analyzed to screen for unexpected effects of this procedural approach. In Germany, ALS response with a specially trained EMS physician is standard, so in both groups, ALS response was carried out with a physician on-scene. According to standardized dispatch criteria, patients requiring ALS (e.g., suspected acute stroke) were either treated by the telemedically equipped ambulance or by a regular ALS unit (Fig. 1). Main dispatch criteria were the availability of the ambulances and units and their distance to the location of the emergency. If the telemedical and standard ALS units had the same distance to the emergency location a primary dispatch of the telemedically equipped ambulance should be conducted. There were no exclusion or inclusion criteria for specific emergencies, so all kinds of ALS missions were dispatched to both groups. Neither the patients nor the EMS physicians or paramedics had any influence on the dispatching, and the dispatch center staff had no knowledge about the study contents. Predefined data were obtained prospectively from all patients who were diagnosed with acute stroke by the EMS physician (including non-traumatic intracranial hemorrhage). When the EMS physician stated “stroke” only as a differential diagnosis, the patient was not included into the analysis. No age restriction or other patient specific exclusion criteria were defined. There was no restriction to a specific time window. The mission was excluded if the location of the emergency was outside the primarily served EMS region. Two stroke centers (one university hospital, one regional hospital) and five community hospitals without stroke units are located in the region served by the EMS.

Originally a randomized controlled trial was planned within this research project and registered (http://www.controlled-trials.com/isrctn/pf/83270177). Due to the restricted availability of the system and the fact that the technical development did not achieve all predefined aims, it was conducted in the described design. The protocol for this trial and supporting CONSORT checklist are available as supporting information; see Checklist S1 and Protocol S1.

### Ethics Statement

Patients were informed about the telemedical data transmission, and informed consent was obtained. The trial was approved by the local ethics committee (University Hospital Aachen, Germany, registration number EK 141/09).

### Telemedicine System and Stroke Specific Approach

**Figure 2:**
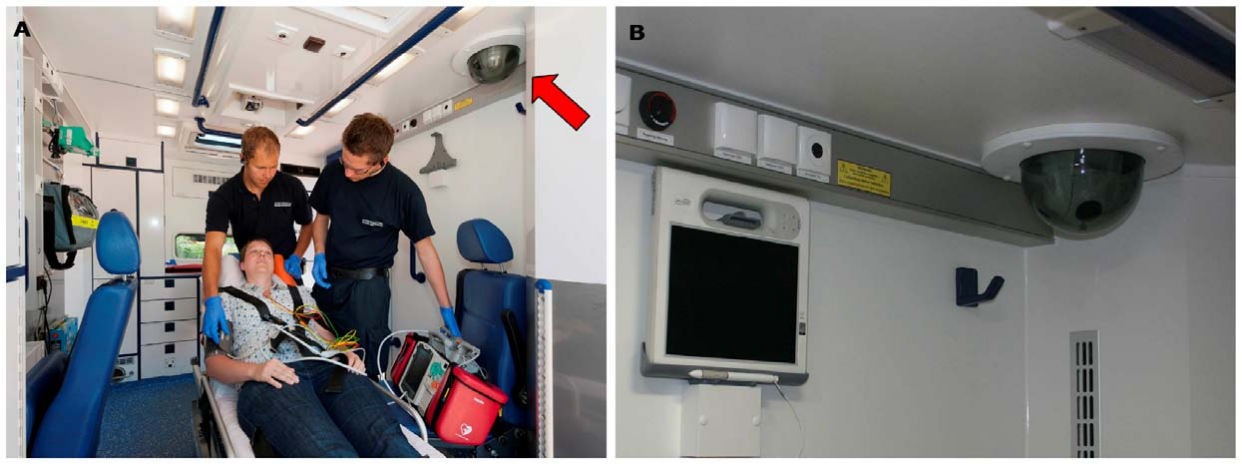
Interior of the telemedically equipped ambulance. Picture A. Trailing scene with a volunteer in the role of a patient and paramedics from the fire department. The video camera is behind a glass cover (Picture B and indicated by the red arrow). The camera position in the ceiling allows zooming to the patient’s face and looking at all body regions from the teleconsultation center. Picture A provided by Peter Winandy, Aachen, Germany.

**Figure 3:**
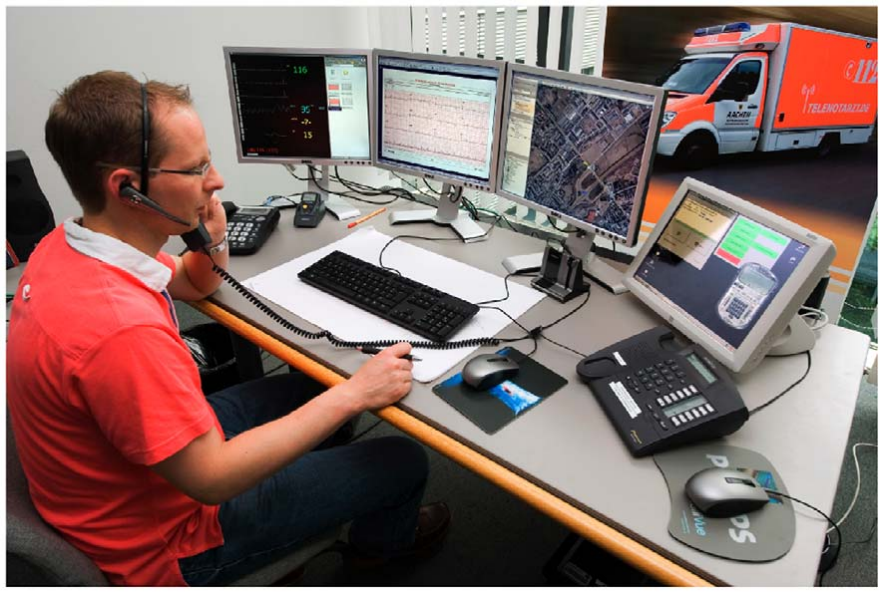
Telemedical workstation. Three monitors display the following information: Vital data (numerical values and curves), transmitted 12-lead-ECGs, transmitted still pictures, video transmission from the ambulance, software to fill out stroke checklist, position of the ambulance via global positioning system, internet access. One touchscreen monitor enabled audio system control and monitoring of data transmission.

The ambulance was equipped with a portable data transmission unit (peeq-box, P3 communications, Aachen, Germany) enabling encoded broadband communication via four parallel data channels from different network providers (each enabled the use of second or third generation mobile networks); max. uplink 1.4 Mbit/s, max. downlink 6 Mbit/s. A vital data monitor (IntelliVue X2, Philips Healthcare, Boeblingen, Germany), a digital camera (Powershot A1000IS, Canon Inc, Tokyo, Japan), three headsets for audio communication (Voyager PRO, Plantronics, Santa Cruz, CA, USA) and a video camera (SNC-RZ 50P, Sony Electronics Inc, San Jose, CA, USA) were connected to the transmission unit. The video camera was embedded into the ceiling of the ambulance and allowed an optical zoom. (Fig. 2). All other components were mobile and could be used inside and outside the ambulance. Audio data, monitoring data, 12-lead ECG, still pictures (4 Megapixel) taken with the digital camera and video streams from inside the ambulance (384×288 pixel (VGA) with 8 frames/second) were transmitted in real time to a teleconsultation center (Fig. 3) staffed with experienced EMS physicians (tele-EMS physicians). Data storage on a secured server was conducted for all data types with the exception of video data, which was not recorded.

**Figure 4:**
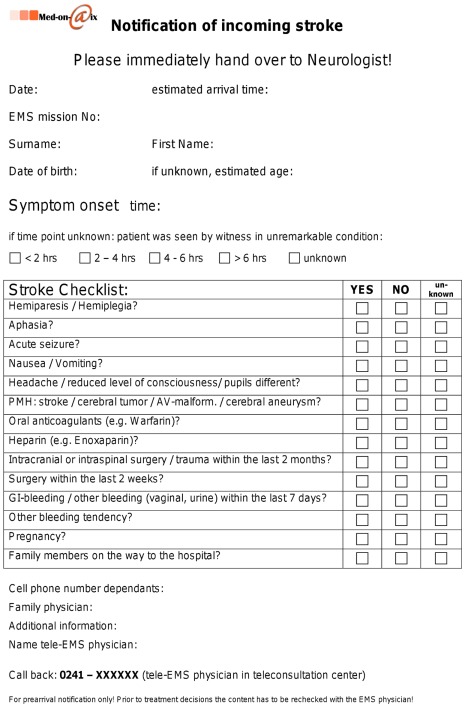
Stroke history checklist used. Translated version, original version in German. The checklist was completed electronically in the teleconsultation center and sent via e-mail to fax to the emergency department and handed over to the neurologist. EMS, Emergency Medical Service; PMH, past medical history.

Stroke specific approach: Continuous two-way audio communication between the EMS team (1 EMS physician and 2 paramedics) and the teleconsultation center was established as soon as possible after arrival at the location of the emergency. The EMS physician immediately took the medical history and examined the patient. Meanwhile, the tele-EMS physician completed a 14-item stroke history checklist (Fig. 4) and supported the ambulance team in medical or organizational issues, if necessary. A committee of local experts consisting of neurologists and EMS physicians created the checklist on the basis of published checklists and recommendations [12], [13], [14]. Vital data was transmitted automatically as soon as the vital data monitor was turned on. Still pictures were taken with the digital camera by the EMS physician, when he attributed an informative value to the content of the picture (e.g., pictures of a medication list, medical report, facial asymmetry). When the patient was transferred into the ambulance, the tele-EMS physician operated the video camera and co-evaluated the neurological status from this remote site. Video streaming was conducted both in the phase while the ambulance was still on-scene and on the way to the hospital (moving ambulance). The tele-EMS physician zoomed to the patient and looked for facial asymmetry, asymmetric body positioning, movements, weaknesses and coordination. He contacted the stroke center for admission capacities and informed them of the incoming patient. The stroke checklist was sent via fax and handed over to the on-call neurologist.

Organizational approach in the control group: According to local routine, the EMS physician on-scene inquired on the admitting capacities of the hospitals via cell phone and conducted pre-arrival notification in the same way. Neither teleconsultation nor transmission of data was performed.

### Personnel

EMS physicians in both groups were board-certified anesthesiologists or at least fourth year residents in anesthesia and critical care and were certified for prehospital emergency medicine. The teleconsultation center and the telemedically equipped ambulance were staffed by a pool of ten specially briefed EMS physicians. The physicians worked equally in both functions (on-scene and tele-EMS physician).

### Objectives and Data Sources

Main objective was to investigate the feasibility of the system and the stroke specific workflow. Furthermore, possible influences on prehospital and initial in-hospital time intervals, the diagnostic quality of the EMS physicians and the transfer of stroke specific data from the prehospital to the in-hospital setting were researched. To evaluate if patient’s characteristics are comparable between both groups, baseline data were obtained from EMS protocol sheets and emergency department (ED) documentation. Time intervals were measured using the time recordings of the EMS dispatch center and the hospital information systems. To evaluate the diagnostic quality of the EMS physicians prehospital and definitive diagnoses were clustered into three categories: (1) stroke – stroke, (2) stroke – different neurological diagnosis, (3) stroke – non-neurological diagnosis. All medical records, EMS protocol sheets and stroke checklists were reviewed to count how many of the 14 stroke-specific items were transferred to the hospital in written form. Standardized questionnaires were used by the tele-EMS physicians to document technical dropouts and to rate qualities of video and still picture transmission as well as their estimated clinical benefit (five-point Likert scale). Qualities of video streaming were rated as a cumulative assessment with respect to the particular case, because video streaming was conducted for several minutes. Qualities of transmitted still pictures were rated the same way because mostly more than one still picture was sent if this application was used. An ancillary analysis of in-hospital data (e.g., rate of thrombolysis, neurological status after 24 hours) was conducted to screen for possible unexpected effects of this novel system and is presented as supplementary material in Appendix S1.

### Statistical Methods

Continuous variables are expressed as medians and interquartile ranges (IQR) and differences were analyzed with the unpaired Wilcoxon test. Categorical data are presented as frequencies and percentages and Fisher’s exact test was used to compare proportions. Due to the exploratory nature of the study (pilot study), no alpha adjustment was performed. Thus, p-values <0.05 were considered to be statistically significant. All statistical analyses were conducted using SAS Version 9.2 (SAS Institute Inc., Cary, NC, USA).

## Results

Overall, 939 patients were treated by EMS physicians in the described study period; 289 were treated by the telemedically equipped ambulance, and 650 were treated by regular ALS units. Figure 1 illustrates the trial flow. One patient in the telemedicine group was excluded because the telemedically equipped ambulance served only to transport the EMS physician to a different ambulance.

### Telemedical Approach

In 15 of 18 missions the telemedicine system functioned faultlessly. In three missions, partial dropouts of single applications were observed: In one mission, audio communication was impossible due to a technical failure. Thus, continuous two-way audio communication was conducted in 17 cases. In a second case vital data, still picture and video transmission failed but audio communication functioned faultlessly. In the third case still picture transmission was impossible. No technical problems were documented for 12-lead ECG transmission (n = 6). In 14 (78%) cases, a pre-arrival notification to the hospital was carried out by the tele-EMS physician via transmission of the stroke history checklist (e-mail to fax), and additional information was shared by phone. In the remaining four cases, information sharing was achieved only by phone. The intention to use video transmission was documented in 14 cases. One patient refused consent and in another case, real-time video transmission failed as described above. Hence, neurological co-evaluation by the tele-EMS physician was conducted in 12 cases. In eight of these cases the video quality was assessed as “excellent” by the tele-EMS physician and in the remaining four cases as “good”. The (subjective) clinical value of the video streaming for remote teleconsultation was assessed as “very helpful” in three, as “helpful” in six, as “rather helpful” in two cases and as “not helpful at all” in a single case. In addition, still pictures - taken with the mobile digital camera - were transmitted in 12 missions and the picture quality was rated as “excellent” (n = 3), “good” (n = 6) and “moderate” (n = 1). The clinical value of the transmitted pictures was rated as “very helpful” (n = 6) or “helpful” (n = 6). In most cases, medication lists and older medical records available on-scene were photographed.

### Influences on Prehospital and in-hospital Processes

Telemedicine group vs. control group: The median times from EMS alarm to EMS physician arrival were 5 (IQR 2) minutes vs. 7 (IQR 4) minutes, p = 0.0182. No statistical significant differences in patient characteristics were found between both groups with the exception of the respiratory rate (table 1). One patient in the telemedicine group required antiarrhythmic medication. All other patients in both groups had heart rhythms with no need for antiarrhythmic treatment. In the telemedicine group, 16 patients were transported to facilities with stroke units, whereas two were not. In these two cases, the reason for this approach was documented (wish of the patient, multi-morbidity and severe dementia). In the control group, 45 patients were transported to a stroke center. One patient was transported to a facility without a stroke unit, and no explanatory statement was documented.

**Table 1:** Subject demographics of all included patients.

parameter	Telemedicinegroup, n = 18			Controlgroup, n = 46			
	n	median	IQR	n	median	IQR	P-value
age (years)	18	80	13	46	80	13	0.8812
female	11	(61%)		30	(64%)		0.7786
GCS*	18	14.5	5	43	15	2	0.4308
NIBP systolic* (mmHg)	18	148.5	43	46	160	60	0.0848
heart rate/min*	18	81	30	45	80	20	0.3525
respiratory rate/min*	15	16	5	20	14	3	0.0012
SpO_2_ * (%)	17	97	6	44	96	2.5	0.3557
blood glucose* (mg/dl)	14	113	35	41	116	55	0.8241

Data are presented as medians and interquartile ranges (IQR). EMS-P, emergency medical service physician; GCS, Glasgow Coma Scale; NIBP, non invasive blood pressure; SpO_2_, oxygen saturation measured with pulseoxymetry;

* initial measurements on scene.

No significant differences were found for the measured prehospital time intervals and the ‘door to brain imaging’ interval for all patients with the prehospital diagnosis of (suspected) stroke (table 2). Also in the subgroup of patients with confirmed stroke no significant differences regarding these time intervals were detected (Appendix S1).

**Table 2:** Prehospital and in-hospital time intervals.

	telemedicine group			control group			
time interval (min)	n	median	IQR	n	median	IQR	P-value
on-scene time	18	25	9	42	21	9	0.1851
contact to hospital arrival	18	37.5	14	41	35	14	0.9671
door to brain imaging*	16	59.5	67.5	42	57.5	80	0.6447

Data are presented as medians and interquartile ranges (IQR).

* beginning of cerebral CAT scan/perfusion MRI.

The comparison of the prehospital diagnostic quality revealed that the prehospital diagnosis of stroke was confirmed in the hospital in 11 (61%) vs. 30 (67%) cases, other neurological diagnoses were found as the definitive diagnosis in 4 (22%) vs. 10 (22%) and non-neurological diagnoses in 3 (17%) vs. 5 (11%) patients, p = 0.8451.

In the telemedicine group, significantly more stroke-specific data were transferred from the EMS to the hospital: a median of 14 (IQR 9, n = 18) vs. 5 (IQR 2, n = 46) items were available in written form, p<0.0001. No major effects or other significant differences between the groups regarding the use of thrombolytics in cerebral ischemia (3/10 vs. 5/27, p = 0.655) or other in-hospital parameters were found in the ancillary analysis (Appendix S1).

## Discussion

The feasibility and effects of prehospital teleconsultation in acute stroke were investigated. The technical performance and reliability of the system were not completely satisfactorily, but the feasibility of both, this system and our stroke specific approach was demonstrated. In comparison with regular care in our EMS no detectable influences or other major effects on clinical processes were found. However, the transfer of stroke-specific data from the prehospital to the in-hospital setting was significantly improved with telemedicine, and a clinical benefit was attributed to video and still picture transmission.

To our knowledge, this is the first study that evaluated prehospital teleconsultation including real-time video transmission from an ambulance in real stroke patients. In one previous study, simulated stroke scenarios were used to evaluate video transmission from an ambulance allowing remote neurologic evaluation, and shortened treatment intervals were found [9]. In contrast to stroke, the benefit of 12-lead ECG transmission with consultation of a cardiologist in acute coronary syndromes is clearly proven, but this telemedical approach does not require a mobile broadband data connection and is now much easier to realize with commercial devices [15], [16], [17], [18].

The technical performance can only be called “satisfactory for this state of development” and improved compared to an earlier observational preliminary study [19], but still three partial dropouts were observed. Temporary dropouts and local non-availability of mobile networks are factors that cannot be influenced by the user. Overload due to a high data traffic in mobile data networks are well known problems and sometimes can slow down transmission times or can even make a data transmission impossible. Especially partial dropouts of video transmission from a moving ambulance are well explicable, because this application needs a stable broad band connection and a moving ambulance changes the network cells rapidly. With the described non-commercial system that uses parallelized data channels great efforts were made to solve such problems and to increase the availability and the data uplink of mobile networks. However, also dropouts due to technical instability within the system were found. Before such a system can be implemented into routine care – and especially to support non physician staffed EMS teams – the technical performance and reliability have to be improved. Because the qualities of the video and picture transmission were rated high and the assessed benefit of transmitted still pictures was also rated high, there is a future potential for these applications. Regarding video transmission, more heterogeneous assessments were observed. However, 75% of the tele-EMS physicians rated this application as “helpful” or “very helpful” from their perspective. At this point it should be mentioned why no vascular neurologist was directly consulted from the EMS team. The teleconsultation system was designed to offer telemedical support in all kinds of emergencies, and it was not possible to hold several specialists available for this function. Moreover, most of the vascular neurologists are not experienced in prehospital emergency care and the system was not only designed to enable a remote diagnosis but real-time medical support regarding all aspects during an emergency mission (e.g., cardiovascular complications). However, a vascular neurologist would probably uprate the clinical value of video transmission and improve the diagnostic quality, especially if the on-scene teams were not physician staffed. Therefore, in the future it might be beneficial that a vascular neurologist could also have parallel access to the video streaming and vital data.

Although all physicians of the telemedicine group were briefed to use the stroke history checklist, compliance was not satisfactorily; pre-arrival notification with the stroke history checklist was realized only in 78%. Similarly, video transmission was not performed in four cases, and explanatory documentation was missing. This shows that the introduction of new, complex technical devices and workflows require intensive training, because even within a small group of participating physicians it was obviously hard to become familiar with this novel function. The fact that only one patient refused consent to video transmission indicates that most patients were unprejudiced to this new technology.

Baseline data of both groups were comparable although two parameters differed significantly. A median difference of two minutes was found for the alarm to arrival time, but in acute stroke (in contrast to e.g. cardiac arrest) such a difference seems to be of minor medical relevance, although it was statistically significant. Furthermore, the respiratory rates differed but the medians of this parameter were within normal (physiological) limits in both groups (table 1). The telemedical approach did not influence prehospital time intervals but on-scene times in both groups were longer than recommended and future efforts must be made to reduce these times. Currently, a maximum of 15 minutes is advised [14]. However, the contact to hospital times were comparable to a different German region [20] and were even slightly shorter in comparison with data from another prehospital stroke research project [21]. But it has to be stated that different transport times due to regional traffic conditions are not influenceable by the EMS team. Potentially harmful time consumptions were found concerning the door to brain imaging times. They are judged to be too long and there is an urgent need for shortening of this interval in both groups. In the group of ‘all patients’, longer median door to brain imaging intervals can be explained with non-stroke diagnoses stated at arrival in the ED by the neurologist (stroke mimics). Therefore, brain imaging might not be performed urgently (e.g., seizure without complications). In the subgroup of “confirmed strokes”, these time intervals were shorter but still too long for acute stroke and have to be improved (Appendix S1). No special training program for neurologists and ED personnel was conducted prior to this study; however, the hospital staff was informed about the telemedicine approach and the new stroke history checklist in lectures. In contrast to many clinical trials, the fact that most of the in-hospital personnel were not aware of the measuring can explain such findings that represent clinical routine. Both stroke centers had several neurologists who received only a few patients notified via telemedicine compared to many “regular patients”. It is understandable that the few study patients did not influence the routine procedures of the stroke centers. If this telemedical approach would be implemented into daily routine, the chance of improving in-hospital processes would emerge. But even in the first phase of the inter-hospital telestroke project TEMPiS, only 74% of the acute stroke patients (onset ≤3 hours) received brain imaging within the first hour after arrival in the study group [22]. Chatterjee et al. found median times from triage to completion of brain imaging of 30 (IQR 18-59) minutes in patients with symptoms <3 hours and 102 (IQR 48-164) minutes in patients with an onset of symptoms >3 hours [23]. Our collective was mixed with patients with an onset of symptoms <3 and >3 hours. Therefore the detected door to brain imaging intervals seem to be no exceptionally unusual finding, but this must not be a justification and the need for improvement is obvious.

The prehospital diagnostic accuracy was at the same level in both groups. The detected proportion of non-stroke diagnoses was comparable to the results of Harbison et al., who analyzed the diagnostic accuracy of primary care physicians, ED physicians and paramedics [24]. Although both groups in our study were physician staffed, the rate of non-stroke diagnoses was comparable to paramedics using the face-arm-speech test [24]. In reading these results, it is important to consider that an over-triage of 30% is volitional and recommended by the American Heart Association to capture all stroke patients [14]. In another German prehospital stroke research project where highly skilled vascular neurologists supported the EMS physician on-scene the diagnosis of stroke was confirmed in the hospital in 72% [21]. In this study also patients with stroke as a differential diagnosis were included which may explain the findings, but even when experienced neurologists are sent to the patient a ‘stroke diagnosis’ cannot be stated reliably in all patients in the prehospital setting. Overall, no harm is to be expected from the determined over-triage.

The considerable better availability of stroke-specific data in the telemedicine group has the potential of treating more patients with thrombolytics if this approach would be trained and implemented into routine care. Some patients are not alert when arriving at the ED and a relative is often not reachable. Checklists with medical information, as well as contact information about relatives and the general practitioner can close this information gap. On the EMS protocol sheets, the Glasgow Coma Scale and the pupil reaction were the only stroke-specific information that could be documented with checkboxes. All other information had to be handwritten in free text. Therefore, a higher amount of missing data in the control group can be explained.

In the ancillary analysis of in-hospital data high rates of thrombolysis were determined, but looking at the small numbers this could be also a purely statistical effect. In Europe, rates of thrombolysis of 10% and higher are reported frequently [20], [25], [26], [27]. In contrast, the rate of thrombolytic use in the U.S. varies between different regions, and only 0.9% of the hospitals reported treatment rates above 10% [28]. The average rate is approximately 5% [29], [30].

The medical standard in the control group was considerably high compared to many other EMS. The ALS response was always carried out by physicians with a minimum requirement of three years experience in anesthesia and critical care, which is considerably above the national requirement. If the comparison of telemedicine vs. regular EMS would be conducted in an EMS with lower qualification levels the availability of expert knowledge may improve the quality of care, especially if in addition a vascular neurologist can be consulted in cases of suspected stroke.

### Limitations

Because of the small sample size, this study may not be adequately powered to detect differences between the groups. Therefore, especially outcomes like the rate of thrombolysis and length of stay in the hospital were only included in an ancillary analysis (Appendix S1). These data have to be interpreted only as supporting information. But the comparison with regular EMS allows the interpretation that no major negative effects could be observed and therefore we did not develop new hypotheses through our observation. Due to the intensive conversation about symptoms and medical history, a true independent, second assessment via video transmission was not possible. Therefore, a comparison of the diagnoses of the on-scene EMS physician and the tele-EMS physician was not meaningful. Consequently this study cannot answer the question whether stroke symptoms can be recognized reliably via mobile video consultation. The physicians in the telemedicine group were briefed to use and fill out the stroke history checklist, and this surely influenced the amount of stroke-specific in-hospital data in this group. However, the checklist was not confidential, and the implementation of the telemedicine approach might have also influenced the procedures of the control group. No data were obtained in patients who were transported with a different prehospital diagnosis but were subsequently diagnosed with stroke in the hospital. The ten physicians in the telemedicine group may not reflect a standard group. But in studies on telemedicine approaches, the group of specialists that are consulted is often small. In the STRokE DOC trial, there were only three vascular neurologists that were consulted [3].

### Conclusions

Multifunctional prehospital teleconsultation in acute stroke was feasible but the technical performance has to be improved prior to a routine use. A better transfer of stroke-specific information to the hospital was achieved, and transmission of stroke history checklists exhibits a potential for improved work processes in the initial in-hospital phase. Shortened clinical time intervals as well as improved diagnostic accuracy were not found. However, no negative effects were detected and the demonstrated feasibility indicates a potential for telemedically-assisted prehospital care, especially in EMS where no highly trained personnel are on scene. If telemedical approaches are introduced, a structured training of all staff members involved should be conducted.

## Supporting Information

Appendix S1 **Ancillary data analysis to screen for additional effects.**
(PDF)

Checklist S1 **CONSORT checklist.**
(PDF)

Protocol S1 **Trial protocol.**
(PDF)
