# Supplementary material for: Feasibility of Prehospital Teleconsultation in Acute Stroke – A Pilot Study in Clinical Routine
Source: PLoS One. 2012 May 18;7(5):e36796. doi: 10.1371/journal.pone.0036796 (PMC3356340; doi:10.1371/journal.pone.0036796)
Supplement: Appendix S1 — Ancillary data analysis to screen for additional effects. (PDF) [file pone.0036796.s001.pdf]

Supporting information: Appendix S1

Ancillary data analysis to screen for additional effects

- in-hospital data and data of the subgroup of patients with confirmed stroke

Background and Methods

We conducted an ancillary analysis of in-hospital data to observe additional effects of the system and the novel process chain in acute stroke care that might lead to new hypotheses requiring further research. The rates of thrombolytic use (recombinant tissue type plasminogen activator, rtPA), the National Institutes of Health Stroke Scale (NIHSS) after 24 hours and the length of hospital stay (LOS) were investigated. Patients that died during the hospital stay were excluded from the analysis of the LOS. Furthermore, we analyzed the subgroup of patients with "confirmed stroke" (including non-traumatic intracranial hemorrhage). In-hospital treatment of patients with intracranial hemorrhage was carried out by the Department of Neurosurgery, and no NIHSS were measured. The use of thrombolytics, the NIHSS, the definitive diagnoses and the LOS were gathered from the medical records.

Statistical methods

Continuous variables are expressed as medians and interquartile ranges (IQR) and differences were analyzed with the unpaired Wilcoxon test. Categorical data are presented as frequencies and percentages and Fisher's exact test was used to compare proportions. Due to the exploratory nature of the study (pilot study), no alpha adjustment was performed. Thus, p-values < 0.05 were considered to be statistically significant. All statistical analyses were conducted using SAS Version 9.2 (SAS Institute Inc., Cary, NC, USA).

Results

Telemedicine group vs. control group: Cerebral ischemia was detected in 56% (n=10) vs. 60% (n=27) of cases, and non-traumatic intracranial hemorrhage was diagnosed in 6% (n=1) vs. 7% (n=3) of the cases. Other definitive diagnoses were found in 39% (n=7) vs. 33% (n=15) of the cases. The median

NIHSS were 12 (IQR 13, n=10) vs. 5 (IQR 15, n=27), p=0.0996. Table S1.1 displays the prehospital and in-hospital time intervals of the subgroup of "confirmed strokes". In patients with cerebral ischemia rtPA was administered in 3 (30%) vs. 5 (19%) of the cases, p=0.655. Only one of three patients in the telemedicine group received thrombolytics within the recommended time frame of 60 min after arrival [1,2] and in the control group two of five patients were within this time frame. In patient #1 of the telemedicine group (Table S1.2) the decision for thrombolytic therapy was made secondary. In this patient the neurological symptoms improved from arrival to completion of brain imaging but then worsened again considerably after cerebral imaging and the decision for thrombolysis was made at this time point.

Table S1.1 Prehospital and in-hospital time intervals – subgroup of confirmed strokes

	**telemedicine group**			**control group**			
**time interval (min)**	n	median	IQR	n	median	IQR	P-value
on-scene time	11	24	9	26	21	10	0.5486
contact to hospital arrival	11	37	13	26	34.5	12	0.9469
door to brain imaging*	11	42	76	28	47.5	35.5	0.8149

Data are presented as median and interquartile range (IQR).

* beginning of cerebral CAT scan / perfusion MRI

Table S1.2 Data of thrombolyzed patients

**telemedicine group**			**control group**		
patient	door to brain imaging*	door to thrombolysis†	patient	door to brain imaging*	door to thrombolysis†
#1	29 min	86 min	#1	35 min	48 min
#2	16 min	57 min	#2	39 min	56 min
#3	36 min	77 min	#3	28 min	72 min
			#4	39 min	68 min
			#5	27 min	70 min

Descriptive presentation of data, statistical comparison not meaningful due to the small sample size.

* beginning of cerebral CAT scan / perfusion MRI; † beginning of intravenous thrombolysis

The median NIHSS of patients with confirmed stroke after 24 hours was 11 (IQR 13, n=10) vs. 3 (IQR 10, n=27), p=0.0522. In each group, two patients died within the hospital stay; of these two, one in each group received rtPA. The length of stay in the hospital for all patients were 10.5 (IQR 13.5, n=16) vs. 7 (IQR 9.5, n=44) days, p=0.2689; patients with confirmed strokes stayed for median times of 15.5 (IQR 10, n=10) vs. 8 (IQR 10.5, n=28) days, p=0.1261.

Discussion

The use of the telemedicine system and the stroke specific process chain did not lead to any measurable effects by analyzing the described in-hospital data. There was no statistical difference in the initial NIHSS but the presented difference can be called medically relevant. This difference seems to be purely by chance, because the dispatching by the EMS dispatch center was not different. Both groups received calls to all kinds of emergencies and severities. The qualification levels of the EMS physicians on scene in both groups were comparable and dispatching of the telemedically equipped ambulance to more severe appearing emergencies seems to be unlikely but cannot be ruled out definitely. On-scene times and contact to hospital times in the subgroup of confirmed strokes did not differ from the group of all patients and no differences between the telemedicine and control group were found. This is well explainable, because if the prehospital diagnosis is 'stroke' the EMS team will treat the patient this way, independent from the definitive diagnosis. The door to brain imaging times of patients with confirmed stroke are too long, but a trend towards a shorter time interval can be seen in both groups, compared to the group of all included patients. In the latter group patients were included that probably did not receive an urgent brain imaging after the initial examination by a vascular neurologist in the emergency department (e.g., seizure without complications). However, even in the high urgent patients who received thrombolytics, the time from arrival to the beginning of the brain imaging was longer than acceptable (Table S1.2). The rates of thrombolysis are high, but this can also be a solely statistical effect due to the small sample size. In Europe, rates of thrombolysis of 10% and higher are reported frequently [3,4,5,6]. But on the other hand, there is no indication for a negative influence of telemedically assisted care on this important outcome factor. The door to thrombolysis times exceed the recommend time limit of 60 min in 5 of 8 patients [1,2]. But the described times seem to be not an exceptionally unusual finding, because comparable times are reported in different settings [7]. However, there is an urgent need for shortening this time interval, independent from the

introduction of a telemedical approach. In both groups the median NIHSS after 24 hours improved, but from a medically relevant different initial value. The LOS showed no statistical difference and a trend towards a longer time in the telemedicine group can be explained by the higher initial NIHSS.

Overall these data represent data from clinical routine care. Most of the in-hospital data was gathered in a big university hospital. The need for improvements in the acute in-hospital phase of stroke patients is recognized and process improvements are sought as a result of these findings. It is obvious that a few study patients did not influence the clinical routine in a big hospital but negative effects of the telemedical approach were not found and no completely new hypotheses regarding this approach have to be researched in future. If new work processes should be implemented into clinical routine, a structured training of all staff members involved in the process chain as well as data feedback to the caregivers is necessary to gain successes and improvements. Scholz et al. showed that a periodical structured data feedback about the performance of the caregivers can lead to an improved time management in patients with acute myocardial infarction [8]. Such a concept would also be meaningful for acute stroke.

References in Appendix S1

1. Adams HP, Jr., del Zoppo G, Alberts MJ, Bhatt DL, Brass L, et al. (2007) Guidelines for the early management of adults with ischemic stroke: a guideline from the American Heart Association/American Stroke Association Stroke Council, Clinical Cardiology Council, Cardiovascular Radiology and Intervention Council, and the Atherosclerotic Peripheral Vascular Disease and Quality of Care Outcomes in Research Interdisciplinary Working Groups: the American Academy of Neurology affirms the value of this guideline as an educational tool for neurologists. Stroke 38: 1655-1711.
2. European Stroke Organisation (ESO) Executive Committee: Collective Name: ESO Writing Committee (2008) Guidelines for management of ischaemic stroke and transient ischaemic attack 2008. Cerebrovasc Dis 25: 457-507.
3. Walter S, Kostpopoulos P, Haass A, Helwig S, Keller I, et al. (2010) Bringing the hospital to the patient: first treatment of stroke patients at the emergency site. PLoS One 5: e13758.
4. Ziegler V, Rashid A, Muller-Gorchs M, Kippnich U, Hiermann E, et al. (2008) [Mobile computing systems in preclinical care of stroke. Results of the Stroke Angel initiative within the BMBF project PerCoMed]. Anaesthesist 57: 677-685.
5. van Wijngaarden JD, Dirks M, Niessen LW, Huijsman R, Dippel DW (2011) Do centres with well-developed protocols, training and infrastructure have higher rates of thrombolysis for acute ischaemic stroke? QJM 104: 785-791.
6. Reiner-Deitemyer V, Teuschl Y, Matz K, Reiter M, Eckhardt R, et al. (2011) Helicopter transport of stroke patients and its influence on thrombolysis rates: data from the Austrian Stroke Unit Registry. Stroke 42: 1295-1300.
7. Price CI, Clement F, Gray J, Donaldson C, Ford GA (2009) Systematic review of stroke thrombolysis service configuration. Expert Rev Neurother 9: 211-233.
8. Scholz KH, Hilgers R, Ahlersmann D, Duwald H, Nitsche R, et al. (2008) Contact-to-balloon time and door-to-balloon time after initiation of a formalized data feedback in patients with acute ST-elevation myocardial infarction. Am J Cardiol 101: 46-52.
