# Supplementary material for: Feasibility of Prehospital Teleconsultation in Acute Stroke – A Pilot Study in Clinical Routine
Source: PLoS One. 2012 May 18;7(5):e36796. doi: 10.1371/journal.pone.0036796 (PMC3356340; doi:10.1371/journal.pone.0036796)
Supplement: Protocol S1 — Trial protocol. (PDF) [file pone.0036796.s003.pdf]

UNIVERSITÄTSKLINIKUM AACHEN

Klinik für Anästhesiologie
Univ.-Prof. Dr. Rolf Rossaint
MEDIZINISCHE FAKULTÄT
RWTH AACHEN

Telefax (0241) 80-82406
Telefon Durchwahl (0241) 80-88179

<u>Prüfplan:</u> Forschungsprojekt Med-on-@ix: Evaluation eines präklinischen telemedizinischen Assistenzsystems in einer prospektiven Vergleichsstudie

Hintergrund:

Der Rettungsdienst in Deutschland steht aktuell vor großen Herausforderungen. Allgemeiner Ärztemangel, stetig wachsender Kostendruck bei steigenden Einsatzzahlen und zunehmender Mangel an Notärzten haben bereits zu messbaren Defiziten in der präklinischen Notfallversorgung geführt. Es bedarf neuer Konzepte, um bestehenden und zukünftigen Problemen im Rettungsdienst zu begegnen. Zudem wird eine deutlich veraltete Informationstechnik eingesetzt. Das Notarztsystem muss einerseits eine Modernisierung und Qualitätssteigerung erfahren, andererseits sind deutlichere Nachweise der Leistungsfähigkeit von Nöten. Nur so kann für die Zukunft eine hochwertige Patientenversorgung gewährleistet werden.
Das deutsche Rettungssystem steht immer wieder in der Kritik, vergleichsweise teuer gegenüber Rettungssystemen zu sein, die keinen Notarzt einsetzen. Der wissenschaftliche Nachweis einer generellen Überlegenheit des Notarztsystems gegenüber „Paramedic"-Systemen scheint nicht realisierbar. Denn dazu wären prospektive, randomisierte und doppelblinde Studien notwendig, die allein aus ethischen Gründen nicht möglich sein werden

Vor dem Hintergrund einer Verbesserung der Versorgungsqulität in der präklinischen Notfallmedizin wurde die Projektidee "Med-on-@ix" durch die Klinik für Anästhesiologie am Universitätsklinikum Aachen entwickelt.
Gesundheitstelematische Einzelanwendungen sind seit vielen Jahren in internationalen Notfallmedizinsystemen untersucht und teilweise etabliert worden. Die Wurzeln der präklinischen Telemedizin finden sich im U.S.-Rettungsdienst. Hier ist die Telekonsultation von Ärzten via Sprechfunk seit den Anfängen des professionellen Rettungsdienstes zum Standardverfahren geworden. Diese Vorgehensweise wird als „online medical control/ command" bezeichnet und dient sowohl der Abstimmung medizinischer Maßnahmen als auch der frühzeitigen Information des Krankenhauses. In vielen US-amerikanischen Bundesstaaten ist die fernmündliche Beratung vor der Applikation ausgewählter Medikamente, wie beispielsweise Morphin, verpflichtend. Weiterhin kann so die primäre Versorgungsqualität genauer beurteilt, gesichert und verbessert werden. Zudem werden innerklinische Abläufe durch einen verbesserten Informationsfluss beschleunigt.

Seit einigen Jahren erfolgt in mehreren deutschen Rettungsdiensten die Übertragung eines 12-Kanal-EKG an die aufnehmende kardiologische Klinik. International konnte gezeigt werden, dass diese

Vorgehensweise zu einer deutlichen Reduktion der Contact-to-Balloon-Time und der Door-to-Balloon-Time führt. Doch selbst solche innovativen Verfahren erfolgen überwiegend unter Nutzung einer zwar nahezu flächendeckend verfügbaren, aber für große Datenmengen nicht geeigneten Übertragungstechnologie der zweiten Mobilfunkgeneration ohne zusätzliche Verschlüsselungsmechanismen. Allerdings sind auch andere Faktoren, wie Organisationsstrukturen und Feedbackmechanismen für entsprechende Erfolge entscheidend.
Daher wird im Projekt Med-on-@ix ein praxistaugliches telemedizinisches Gesamtkonzept entwickelt. Modernste Übertragungstechnik, Schulung und Weiterqualifizierung des Personals, detaillierte elektronische Dokumentation und moderne Organisationsstrukturen werden in das System integriert. Es werden außschließlich zugelassene Medizinprodukte und Medikamente verwendet. Bereits heute werden im regulären Verfahren der präklinischen Notfallmedizin Konsile (z.B. Herzkatheteranfrage) telefonisch durchgeführt und die Datenübertragung (12-Kanal-EKG) ist gängiger Standard.

Zielsetzung:

Ziel des Projektes ist die Steigerung von Versorgungsqualität für Notfallpatienten. Weiterhin soll dabei die Möglichkeit der Effizienzsteigerung im Einsatz durch Prozessoptimierung in den Blick genommen werden. Ein umfassendes notfallmedizinisches Telemedizinsystem wird entwickelt, das Notärzten und Rettungsdienstpersonal eine additive Beratung durch ein Kompetenzzentrum bereitstellt. Die leitliniengerechte Behandlung soll dadurch unter direkter Qualitätskontrolle sichergestellt werden.

Fragestellung:

Folgende Fragen sollen im Detail geklärt werden:

- Ist eine Verbesserung der notfallmedizinischen Patientenversorgung möglich, wenn der Notarzt im Einsatz durch ein Telemedizinsystem (als reines „Add-on") unterstützt wird?
- Welche Effizienzreserven bestehen im Rettungswesen und lassen sich durch den Einsatz von Telemedizin nutzen?
- Welche Techniken der kommerziellen Mobilfunkinfrastruktur sind zuverlässig zur sicheren und mobilen Echtzeitdatenübertragung einsetzbar und kombinierbar?

Methodik:

Zentrales Element des Systems ist ein Kompetenzzentrum, das mit einem erfahrenen Notfallmediziner (Facharzt Anästhesiologie mit Fachkundenachweis Rettungsdienst oder Zusatzbezeichnung Notfallmedizin; im weiteren „Telenotarzt“ genannt) besetzt wird. Mithilfe weiterentwickelter mobiler Datenübertragung werden in Echtzeit alle (Vital-)Parameter des Patienten sowie Video- und Bildmaterial von der Einsatzstelle und auch Auskultationsbefunde dorthin übertragen. Im Kompetenzzentrum werden diese Informationen übersichtlich auf Bildschirmen dargestellt. Der Telenotarzt unterstützt und berät das vor Ort tätige Team bei der leitliniengerechten Therapie. Dabei nutzt er eine spezielle Software, die aktuelle Behandlungsleitlinien abbildet, eine Dokumentationsfunktion hat und (Wissens-)Datenbanken zur Verfügung stellt. Rettungsleitstelle und Kompetenzzentrum kooperieren als jeweils eigenständige Einrichtungen. Notrufannahme und Disposition der Rettungsmittel bleiben, wie in der derzeitigen Praxis auch, grundsätzlich Aufgabe der Leitstelle. Kontaktaufnahme und Datenübertragung zur Klinik sowie die Rücksprache mit anderen Einrichtungen (Hausarzt, Kardiologie, Giftnotrufzentrale etc.) erfolgen vom Kompetenzzentrum aus. Somit wird das Rettungsteam bei seiner hauptsächlichen Aufgabe, der Patientenversorgung, unterstützt und von organisatorischen Tätigkeiten entlastet. Damit soll eine Qualitätsverbesserung der medizinischen Versorgung bei gleichzeitiger Beschleunigung der Prozesse erreicht werden.

Im Vorfeld des Projektes haben zwei Rechtsgutachten festgestellt, dass der Einsatz des Telenotarztes im Rahmen des Projektes Med-on-@ix nach den üblichen Regeln des Rettungsdienstes erfolgt und grundsätzlich rechtskonform möglich ist bei aktueller Gesetzeslage. Für die reine Konsultation eines notärztlichen Kollegen ist prinzipiell keine Einwilligung erforderlich. Sollten jedoch weitere Daten übertragen werden, wobei insbesondere die Übertragung von Bildmaterial bedeutsam ist, dann muss der Patient darüber und über etwaige Risiken aufgeklärt werde. Hierbei ist im Rettungsdienst allerdings immer die zeitliche Dringleichkeit der Maßnahmen und die Situation des Patienten zu berücksichtigen. Aus Gründen der Praktikabilität und des eventuell für die Patientenversorgung nachteiligen Zeitbedarfs für eine Aufklärung, entscheidet dabei die konkrete Notfallsituation über das Ausmaß der Aufklärung sowie die Möglichkeit und Fähigkeit des Patient wirksam einzuwilligen – so wie bei jeder weiteren notfallmedizinischen Maßnahme auch. Nach dem Prinzip der minimalen Datenhaltung wird über die fest installierte Kamera im Rettungswagen sämtliches Bildmaterial nur im sog. „Streaming-Verfahren“ übertragen und nicht gespeichert. Hier ist die Kamera folglich nur als „verlängertes Auge“ des konsiliarisch tätigen Telenotarztes zu sehen, das ausschließlich in der konkreten Situation für eine verbesserte Information und Einschätzung zur Verfügung steht. Weiteres Bildmaterial (z.B. Fotos von Medikamentenlisten, Arztbriefen, Tablettenschachteln o.ä. vor Ort) wird unter höchsten technischen Sicherheitsvorkehrungen zusammen mit der regulären Einsatzdokumentation digital gespeichert und vor unbefugtem Zugriff geschützt. Sollte aus Gründen der weiteren wissenschaftlichen Auswertung Bildmaterial darüber hinaus erforderlich sein, wird die Einwilligung des Patienten eingeholt oder das Material soweit technisch nachbearbeitet, dass es anonymisiert ist. Die routinemäßigen Auswertungen finden anhand der Daten statt, die auch im aktuellen Notarztdienst bzw. der weiteren klinischen Versorgung erhoben werden. Weil keine direkte medizinische Intervention erfolgt ist, ist davon auszugehen, dass auch keine nachträgliche Einwilligung des Patienten eingeholt werden muss. Sämtliche Auswertungen dürften bzw. müssen auch zurzeit ohne direkte Einwilligung des Patienten im Rahmen von Analysen des Qualitätsmanagements durchgeführt werden.

Zur datenschutzrechtlichen Komponente des Projektes stellen die Rechtsgutachten fest, dass datenschutzrechtliche Beschränkungen durch den Einsatz des Telenotarztes durch entsprechende Verschlüsselungen, die durch die Konsortialpartner ZLW/IMA und P3-Solutions erarbeitet worden sind, gewährleistet werden.
Der Einsatz von neuen Behandlungsmethoden, Arzneimitteln oder Medizinprodukten ist im Rahmen des Projektes Med-on-@ix nicht vorgesehen.

Probanden

In die Studie werden alle Notfallpatienten ohne Alterseingrenzung eingeschlossen, welche im Rahmen der regulären Notfallrettung der Stadt Aachen versorgt werden. Weitere Kriterien wie Geschlecht, Familienstand oder Versicherungsstatus sind nicht vorgesehen.

Studienablauf

In der Evaluationsphase wird ein Rettungswagen der Berufsfeuerwehr in diesem Zeitraum vollständig telemedizinisch ausgestattet, mit einem Notarzt besetzt und als Notarztwagen (NAW) in Dienst gestellt sein. Damit wird das System Med-on-@ix bei Realeinsätzen angewandt und evaluiert. Der Einsatz in dieser Phase erfolgt bewusst ausschließlich unter Anwesenheit und Verantwortung eines erfahrenen Notarztes vor Ort, damit maximale Sicherheit für den Patienten und Rechtssicherheit für das Rettungsdienstpersonal gewährleistet sind. Parallel werden eine umfassende Dokumentation und die wissenschaftliche Auswertung von Einsätzen des Regelrettungsdienstes durchgeführt. Eine prospektiv vergleichende Untersuchung der beiden Systeme (Regelrettungsdienst vs. Telemedizinsystem) wird so möglich. Dabei erfolgt eine Art „Pseudo-Randomisierung“, weil sich die Einsätze je nach Zeitpunkt des Anrufes und damit Verfügbarkeit des Telenotarzt-NAW auf die Kontroll- oder Studiengruppe verteilen, ohne dass die Studienleitung einen direkten Einfluss auf diese Patientenzuteilung hätte. Eine aktive Aktion zur Randomisierung, die zu einer direkten Zuteilung der Patienten führt, findet jedoch seitens der Studienleitung nicht statt.

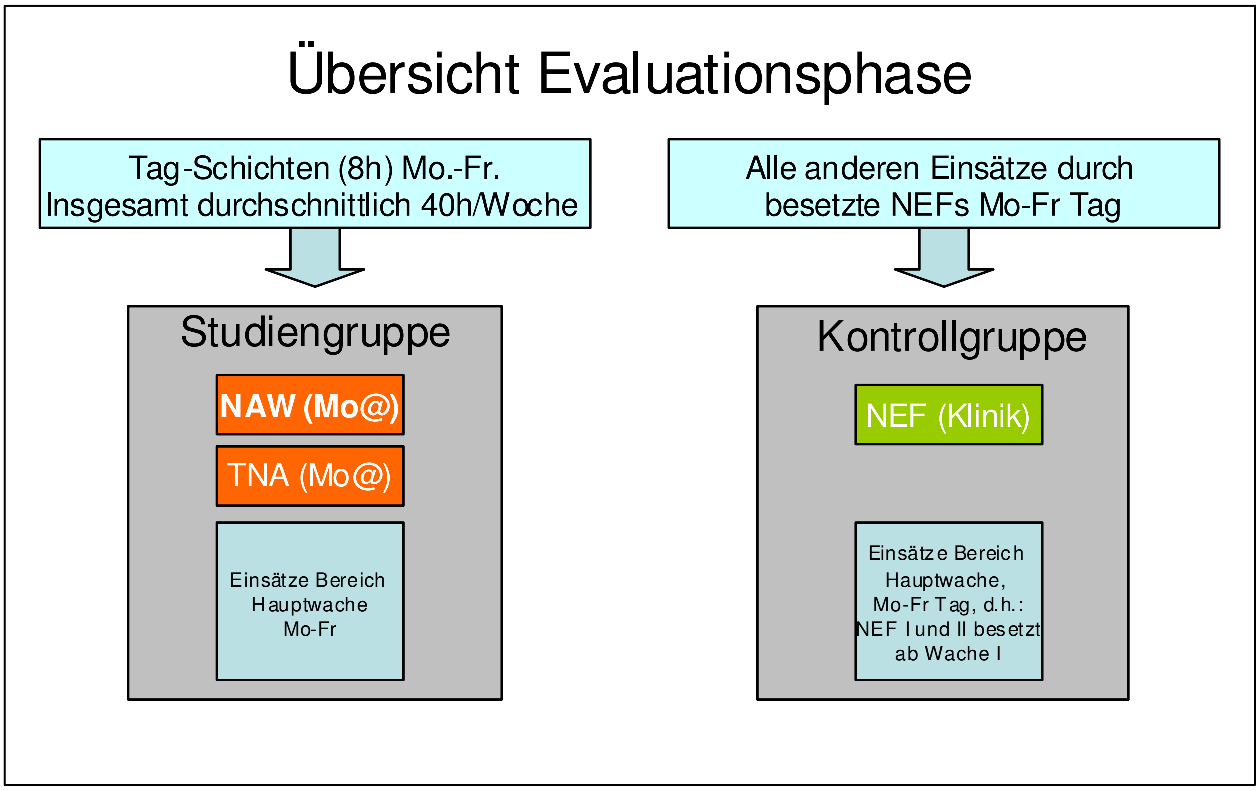

Legende zur Abbildung:

NAW = Notarztwagen, dies ist der Rettungswagen mit dem Med-on-@ix-System, welcher zusätzlich immer mit einem Notarzt besetzt ist

TNA = Telenotarzt

NEF = Notarzteinsatzfahrzeug, bisher auch eingesetzter PKW, der den Notarzt im Rendesvous-System zu den entsprechenden Einsätzen transportiert, bei denen zusätzlich ein Rettungswagen vor Ort ist

Die Evaluationsphase soll mit einer Gesamtlaufzeit von 8 Monaten stattfinden. Zuvor hat bereits eine rein technisch fokussierte Praxiserprobung und Evaluation stattgefunden, bei der keinerlei zusätzliche Datenerfassung oder medizinisch-wissenschaftliche Auswertungen erfolgt sind.

Nach Start der medizinisch-wissenschaftlichen Evaluation sind pro Monat ca. 100 Einsätze zu erwarten, so dass im Zeitraum 01.04.2010 – 30.11.2010 mit ca. 800 Einsätzen, d.h. 400 Patienten pro Gruppe, zu rechnen ist.

Durch die Datenerhebung geht keine Gefahr für die Gesundheit der Studienteilnehmer aus, Nebenwirkungen und Wechselwirkungen sind nicht zu erwarten.

Auswertung:

Im Forschungsprojekt Med-on-@ix soll zusätzlich die *Akzeptanz* des Telenotarztsystems überprüft werden. Dazu wird unterschieden in eine interne Akzeptanzerhebung und die Akzeptanz bei externen Beteiligten. Zu den internen Beteiligten am Telenotarztsystem zählen die Rettungsassistenten und Notärzte, die im Rettungswagen mit dem Telenotarzt kommunizieren. Die Gruppe der Externen, die mit dem Telenotarztsystem im Rahmen des Forschungsprojektes Med-on-@ix in Berührung kommen, ist fallweise größer. Hier kommen nicht nur Patienten und deren Angehörige in Frage, sondern - je nach Einsatzsituation - auch andere Ärzte in der hausärztlichen Versorgung. Von dieser fallweise unterschiedlichen Gruppe von Beobachtern des Telenotarztsystems sollen die Patienten zur Akzeptanz befragt werden, da sie aus dieser Gruppe während der gesamten Einsatzdauer mit dem Telenotarztsystem in Kontakt stehen.

Es soll durch eine kurze schriftliche und/oder telefonische Befragung anhand eines Fragebogens retrospektiv ermittelt werden, ob Patienten das Telenotarztsystem wahrgenommen haben und wenn ja, ob sie das System als irritierend, störend oder auch als beruhigend und hilfreich empfunden haben.

In die Befragung werden volljährige Notfallpatienten eingeschlossen, die zum Übergabezeitpunkt in der Klinik einen nicht eingeschränkten Bewusstseinszustand hatten (Glasgow Coma Scale 15), so dass sie in der Lage gewesen sind, das Med-on-@ix-Telemedizinsystem wahrzunehmen. Ausgeschlossen werden Patienten mit psychiatrischen Erkrankungen und nicht einwilligungsfähige Patienten.

Anhand der Einsatzprotokolle werden die Patienten ermittelt, bei denen die Einschlusskriterien vorliegen. Diesen wird der Fragebogen auf dem Postweg zur Beantwortung zugestellt. Die Rücksendung erfolgt anonym, ebenso die Auswertung. Lediglich bei Ermittlung der Patienten, welche die Einschlusskriterien erfüllen, wird eine Zuordnungsliste mit einem ID-Code für die Fragebögen erstellt. Sollte die Rücklaufquote der schriftlichen Befragung so gering sein, dass aussagekräftige und valide Schlussfolgerungen nicht möglich sind, soll eine telefonische Kontaktaufnahme mit – bei Zustimmung des Patienten – standardisierter Befragung erfolgen.

Für die Evaluationsphase des Forschungsprojektes Med-on-@ix sind keine Abbruchkriterien erforderlich, da der Studie eine Testphase zur Technikoptimierung vorgeschaltet ist. Bei möglichen Fehlfunktionen oder technischem Versagen des Telekonsultationssystems kann jederzeit unverzüglich auf die regulären

Technologien und die üblichen Vorgehensweisen zurückgegriffen werden, ohne dass hierdurch in irgendeiner Weise Nachteile für den Patienten zu erwarten sind.

Ein weiterer Baustein des Forschungsprojekts Med-on-@ix stellt die Überprüfung des Einflusses des Systems auf die *Dokumentationsqualität* dar. Hierbei soll konkret überprüft werden, welchen Einfluss die Beobachtung des Einsatzes und die dabei erhobenen Daten durch den Telenotarzt haben. Die Unterstützung des Telenotarztes bei der Erhebung von entscheidungsrelevanten Daten soll anhand der Einsatzszenarien bewertet werden.

Mithilfe der im Forschungsprojekt Med-on-@ix erhobenen Daten soll Aufschluss über die Inanspruchnahme des Telenotarztsystems in Aachen geben werden. Speziell sollen hierbei Einsatzindikationen und Verletzungsmuster analysiert werden. Neben der Einschätzung zur Häufigkeit auftretender Krankheitsbilder bzw. der im Einsatz angetroffenen Verletzungen kann eine Beschreibung der Auslastung des Telenotarztsystems vorgenommen werden.

Der Telenotarzt in der Kompetenzzentrale hat durch seine Ausstattung mittels PC-Unterstützung und weiterer Literatur einen Überblick über aktuelle und einsatzrelevante *Leitlinien*. Im Rahmen des Forschungsprojektes soll der Einfluss des Telenotarztes auf eine leitlinienkonforme Behandlung erhoben werden. Dazu sollen Einsatzdokumentationen mit aktuellen Leitlinien abgeglichen werden. Die dabei im Hintergrund zu überprüfende Forschungsfrage besteht im Einfluss des Telenotarztes auf eine leitlinienkonforme Behandlung. Der Telenotarzt wird im besten Fall den am Einsatzort befindlichen Notarzt bei der Abarbeitung der Inhalte der Leitlinie unterstützen und dazu sollen die Vorteile und eventuell auch auftretenden Hindernisse analysiert werden.

Das Forschungsprojekt Med-on-@ix wird sich der Beurteilung der *Diagnosequalität* widmen. Dabei steht im Vordergrund die Einschätzung der Situation durch den Telenotarzt und den am Einsatzort befindlichen Notarzt in der Analyse und Bewertung anhand des weiteren klinischen Verlaufs. Dazu soll in den die Patienten aufnehmenden Krankenhäusern eine Nachrecherche der Diagnosen vorgenommen werden, um diese mit der Verdachtsdiagnose in der präklinischen Notfallmedizin zu vergleichen.

Im Zusammenhang mit der Bewertung der Diagnosequalität soll auch die Krankenhausverweildauer in den Blick genommen werden. Eine im klinischen Verlauf bestätigte Verdachtsdiagnose wird zum schnelleren Gesunden des Patienten beitragen und im Idealfall kann die zur Vergleichsgruppe erhobene Krankenhausverweildauer sinken. Diese Erhebung erfolgt nicht als primäres Ziel des Forschungsprojektes Med-on-@ix, aber die Auswertungen der Daten soll sich der ökonomischen Dimension nicht verschließen. Eine sensible Interpretation ökonomischer Daten ist den am Forschungsprojekt Med-on-@ix Beteiligten bewusst.

Eine Archivierung und der Zugriff auf die in der Einsatzsituation entstehenden Daten (Fotos und Audio, d.h. die Gespräche zwischen Notarzt und Telenotarzt) erfolgt nach dem Grundsatz der „Datenvermeidung und Datensparsamkeit“ im Sinne des § 3a BDSG. Bewegte Bilder der Kamera im Fahrzeug werden überhaupt nicht gespeichert, Fotos werden – wie oben beschrieben – besonders geschützt zusammen mit der Einsatzdokumentation gesichert und Audiodaten werden – für den Fall kurzfristiger Rückfragen – sieben

Tage lang archiviert. Nach Ablauf dieser Zeitspanne werden die Aufnahmen automatisiert gelöscht und eine weitergehende Sicherstellung des Zugriffs auf die Daten ist nicht erforderlich. Die kurzfristige Speicherung erfolgt nicht aus Gründen der weiteren Behandlung des Patienten und hat auf diese keinen Einfluss. Sollte aus Gründen der weiteren wissenschaftlichen Auswertung Bildmaterial darüber hinaus erforderlich sein, wird die Einwilligung des Patienten eingeholt oder das Material soweit technisch nachbearbeitet, dass es anonymisiert ist. Eine Bereitstellung personenbezogener Daten an unbefugte Dritte findet selbstverständlich nicht statt, somit verbleiben die Daten im Kreis der an der Patientenversorgung beteiligten Forscher.

Das Forschungsprojekt ist auf die Erprobung eines verbesserten Systems der Notfallversorgung angelegt. Dazu gehört die organisatorisch-logistische Optimierung der gesamten Rettungskette. Um einerseits Schaden durch die Anwendung nicht validierter Behandlungsmethoden vom Notfallpatienten abzuwenden, ihm andererseits aber nicht ein möglicherweise überlegenes Verfahren vorzuenthalten, müssen sowohl in einer medizinrechtlichen als auch in medizinethischer Bewertung Nutzen und Risiken des jeweiligen Forschungsprojektes sorgfältig gegeneinander abgewogen werden. Das Vorhaben Med-on-@ix verspricht neue Erkenntnisse in der präklinischen Notfallmedizin, so dass das verfolgte Forschungsziel der schrittweisen Verbesserung der Notfallversorgung ein vorzugswürdiges Gut gegenüber der engen Auslegung der Privatsphäre des Patienten darstellt.

Mit der im Forschungsprojekt vorgesehenen bedarfsweisen Speicherung und Auswertung von Daten wird einem individualethischen Bedürfnis nach der begrenzten Verwendung der Daten und Informationen Rechnung getragen ohne eine Überlegung nach einem Nutzen für eine Gruppe unzulässig den Vorrang zu geben.

Abschließend sei nochmals betont, dass grundsätzlich sämtliche Möglichkeiten zur Verfügung stehen, wie im regulären Rettungs- und Notarztdienst auch. Das Telekonsultationssystem steht hier für Evaluationszwecke zusätzlich zur Verfügung und kann jederzeit auf Entscheidung des verantwortlichen Notarztes vor Ort außer Acht gelassen werden.

Beteiligte:

Ärzte der Klinik für Anästhesiologie, Universitätsklinikum Aachen

Dr. med. Max Skorning (Hauptprüfer)
Dr. med. Stefan Beckers
Dr. med. Sebastian Bergrath
Dr. med. Daniel Rörtgen
Dr. med. Jörg Brokmann (Ärztlicher Leiter Rettungsdienst)
Harold Fischermann
In-Sik Na
Univ.-Prof. Dr. Phil. Dipl.-Psych. Siegfried Gauggel (beratend)
Heiko Schmitz M. A.

Prüfklinik:

Klinik für Anästhesiologie
Univ.- Prof. Dr. Rolf Rossaint
Universitätsklinikum Aachen
Pauwelsstraße 30
52074 Aachen

Dr. med. Max Skorning

UNIVERSITÄTSKLINIKUM AACHEN

Klinik für Anästhesiologie
Univ.-Prof. Dr. Rolf Rossaint
MEDIZINISCHE FAKULTÄT
RWTH AACHEN

Telefax (0241) 80-82406
Telefon Durchwahl (0241) 80-88179

English Translation of the Study Protocol of the Med-on-@ix Project

Study protocol: Research project Med-on-@ix

Evaluation of a prehospital telemedical assistance system in a prospective, controlled trial

Background:

The German Emergency Medical Service (EMS) actually faces great challenges. General shortage of physicians, increasing cost pressure accompanied by an increasing number of emergency missions and a shortage of Emergency Medical Service physicians (EMS physicians) already led to measurable deficits in the emergency medical care. New concepts are needed to solve the current and future problems in Emergency Medical Services. Furthermore an overaged information technology is used. The EMS physician system has to be modernized on one hand but on the other hand better evidence of its effectiveness is needed. Only in this way a high standard in patient care can be guaranteed in future. The German EMS is frequently criticized to be very costly compared to EMS systems, that do not send a physician on-scene. The scientific prove of a general superiority of a physician powered EMS over a Paramedic staffed EMS seems to be not realizeable. To achieve this goal, prospective, randomized, double blinded trials would be necessary and such trials would probably not be approved because of ethical concerns.

Against the background of an improvement of the quality of care in prehospital emergency medicine, the project Med-on-@ix was developed by the Department of Anesthesiology of the University Hospital Aachen. Single telematic applications are researched since many years in international emergency systems and are transferred to clinical routine sometimes. The roots of prehospital telemedicine are to find in the U.S. EMS systems.

Consultation of emergency physicians via radio has become a routine procedure since the beginning of professional EMS systems in the U.S. This approach is also called „online medical control/command“ and is used for consultation about medical procedures and early notification of the hospital as well. In many states of the U.S. consultation of a physician via radio is mandatory prior to application of certain iv-medications, e.g. Morphine. Additionnallly, the primary quality of care can be assessed, secured and improved. In-hospital processes can be enhanced due to an inproved flow of information.
In some german EMS the transmission of the 12-lead-ECG is carried out since some years. International research indicates that this approach leads to a significant reduction of the „contact-to-balloon time“ and „door-to-balloon time“. But such innovative approaches are conducted with the use of second generation mobile networks without encryption. However, other factors like organizational aspects and feedback mechanisms are crucial for success. Against this background a telemedicine system specifically designed for medical practise is developed. Modern transmission technology, training and qualification of the personnel, electronic documentation and modern organizational structures are implemented and integrated into the system. Only approved standard medical devices and medications will be used. Today pre-notification of the hospital via mobile phone and transmission of the 12-lead-ECG is clinical standard.

Aim:
Aim of the project is the improvement of the quality of care for emergency patients. Another focus is the possibility of an improvement of the efficiacy due to improvements of processes. A comprehensive telemedicine system will be developed, that allows a additive consultation for physcians and paramedics as well. Guidelines conform treatment should be secured with real-time quality control.

Objective :
The following objectives should be researched in detail:

- Will the „add-on“ support for the EMS physician with a telemedicine system lead to an improved medical treatment of patients?
- Which reserves of efficiency are to find in the EMS and can be used with the use of telemedicine?
- Which technologies of the consumer mobile networks can be used for safe and reliable real-time data transmission and can be combined?

Methods:

Central core element of the system is a competence center that is staffed with physicians expirienced in emergency medicine, called "tele-EMS physicians" (board certified Anesthesiologists, certified for prehospital emergency medicine). With the use of further developed data transmission all vital parameters of the patient including still pictures and videostreaming are transmitted from the scene of the emergency to the competence center (including auscultation data). These information are displayed in the competence center on standard display monitors. The tele-EMS physician supports the EMS team that is on-scene with therapy recommendations in conformity to guidelines. Therefore he uses specific software, that displays current guidelines and enables documentation as well access to medical databases. The EMS dispatch center and the competence centre cooperate but stay individual facilities. Emergency calls and disposition of the ambulances are carried out by the EMS dispatch center – like in daily routine – while consultation of e.g. family physicians and pre-notification of the hospital are conducted by the competence center. Thus, the EMS team is relieved of this organizational tasks and can focus on the treament of the patient. With this approach an improvement and enhancement of the process chain of medical treament should be realized.

Prior to the project two legal opinions were obtained. These opinions have determined, that runnning of the system within the project Med-on-@ix is in conformity with the standards in EMS and is in accordance with the current legal situation. For the pure verbal medical consultation of a colleague (physician), no consent of the patient is required in principle. However, if additional data should be transmitted, in particular the transmission of video, then the patient has to be informed about potential risks. In this connection the time urgency and the current situation of the patient (severity of the emergency) are always to consider. For reasons of practicability and the potential negative effect of a time consuming patient information, the information and education of a patient will be adapted to the specific case and the severity of emergency. The severity of the emergency determines the ability of the patient to give an effective consent, like in every other medical procedure, too. Following the principle of minimal data storage, the video camera in the ambulance will only be used with streaming technology and videos will not be recorded and stored. Consequently, the video camera is like an extended eye of the consulting tele-EMS physician, which should enable improved information and assessment by the tele-EMS physician. Still pictures (e.g., still pictures of medication lists, medical documents, drugs and medications) will be saved and stored digitally with the highest possible standard of encoding. Thus, data is protected against unauthorized

access. If further pictures are required for reasons of scientific evaluation, the informed consent of the patient will be obtained or the pictures will be edited that they are completely anonymized. The planned scientific evaluation will be conducted with data that are currently also collected in the standard EMS and in in-hospital processes. Because of the fact, that no medical (invasive) intervention will be performed, no belated informed consent by the patient has to be obtained. All evaluations should or must be currently carried out without the direct consent of the patient in the context of analyses within quality management systems. For the data protection component of the project the legal opinions determined that data-protection issues can be guaranteed by appropriate encodings that are realized by the consortium partners ZLW/IMA and P3 Solutions. The use of new treatments, drugs or medical devices is not intended within the project Med-on-@ix.

Patients

In the trial, all emergency patients will be enrolled irrespective of age, that are treated within the regular EMS care of the city of Aachen. Other criteria for inclusion or exclusion like sex, marital status or type of medical insurance are not defined.

Trial

Within the evaluation and trial phase one mobile intensive care unit of the fire department will be equipped with the telemedicine system and will be staffed with an EMS physician. Thereby, the system Med-on-@ix will be used in real emergencies and will be evaluated. The utilization of the system in this phase is intentionally conducted only by experienced EMS physicians on-scene, ensuring a maximum of patient safety and legal certainty for the paramedics. In parallel, a comprehensive documentation and scientific evaluation of the regular EMS missions will be carried out. A prospective, controlled evaluation of both systems (regular EMS vs. telemedically supported EMS) is possible this way. A kind of pseudo-randomization takes place, because the missions will be assigned to the telemedicine group and control group on the basis of the time point of the emergency call and current availability of the ambulances. The study personnel have no influence on the dispatching of the ambulances and allocation to the groups. An active procedure to randomize patients is not carried out by the project's staff.

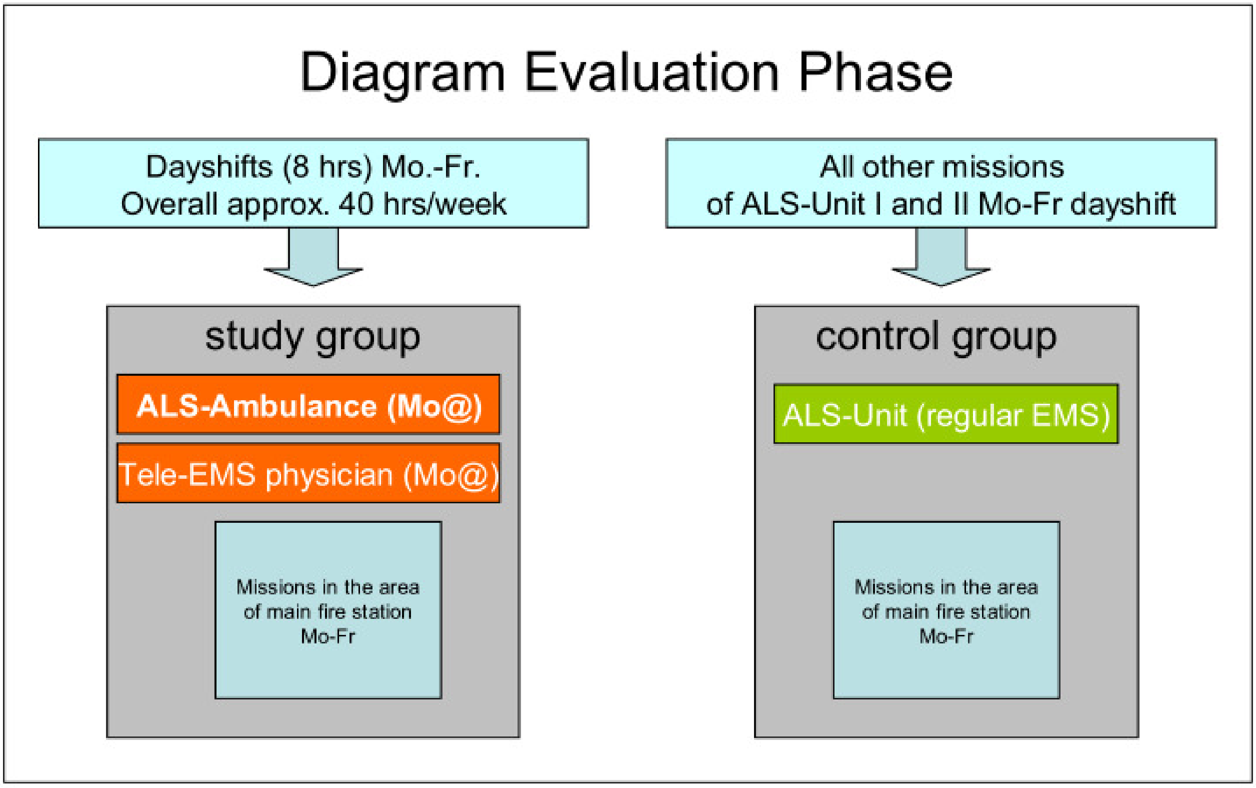

Legend:

ALS-Ambulance	= Ambulance, staffed with an EMS physician and paramedic
Tele-EMS physician	= EMS physician at remote site (competence center)
ALS-Unit	= ALS response car, staffed with EMS physician and paramedic, meets an ambulance on-scene

The study phase is planned for a time interval of 8 months. Prior to this an evaluation phase focussed on technical measures is conducted. No additional data collection or medical evaluation are carried within this pilot phase. We estimate the utilization of the EMS with 100 emergency missions per month. Thus, approximately 800 missions (400 missions / patients in each group) will be conducted between 1 April, 2010 and 30 November, 2010.

Evaluation:

Within the research project Med-on-@ix the acceptance of the telemedicine system should be evaluated additionally:

A distinction is made between an internal survey acceptance and external acceptance of patients. Internal acceptance: EMS physicians and paramedics who communicate between the ambulance and the tele-EMS physician will be questioned. The group of external people that have contact with the system is occasionally bigger. Not only patients and relatives but also family physicians are involved. Of this external group, the patients

should be questioned about their acceptance of the telemedicine system. With a short questionnaire or a short interview by Telephone, it should be analyzed retrospectively, if patients were aware of the telemedicine system and if the system was perceived irritating, disturbing or helpful. In the survey only adult emergency patients (18 years or older) will be included who were alert and in a non-restricted state of consciousness (Glasgow Coma Scale 15) at the time point of the hospital arrival, so that they have been able to perceive the Med-on-@ ix telemedicine system. Patients with psychiatric diseases and patients who were not able to give informed consent are excluded from this survey. Based on the EMS protocol sheets, patients are identified who meet the inclusion criteria. A questionnaire will be send by post to these patients. The return will be anonymously, just as the evaluation. Only in determining the patients who meet the inclusion criteria, a list of assignments is created with an ID code for the questionnaires. If the return rate of mail survey should be so low that no meaningful and valid conclusions are possible, a telephone contact with a standardized consultation will be carried out - with the consent of the patient.

For the evaluation phase of the research project Med-on-@ix no termination criteria are necessary because the study is preceded by a pilot phase for the optimization of the technical system. For any malfunction or technical failure of the teleconsultation system it can always be immediately resorted to the usual regular technologies and processes of EMS, without an expected disadvantage for the patient.

A further element of the research project Med-on-@ix is the evaluation of the influence of the system on the documentation quality. It should be assessed specifically, which influence the observation of the mission by a tele-EMS physician has. The support by the tele-EMS physician in the collection of crucial data should be evaluated in terms of operational emergency scenarios. With the help of the data collected within the research project Med-on-@ ix the utilization of the system in Aachen is measurable. Especially the emergency indications and severities of injuries should be analyzed. Beside the assessment of the incidence of certain emergencies a representation of the utilization of the telemedicine system can be conducted.

The tele-EMS physician in the competence center has an electronic (computer-based) access to current and relevant guidelines. The influence of the system the medical treatment and its conformity to current guidelines should be analyzed. Therefore, medical documentation will be compared to current medical guidelines. The objective is if the tele-EMS physician has an influence on the treatment processes and if this influence is in

accordance with current guidelines. The tele-EMS physician will assist the EMS physician on-scene with the contents of guidelines. The potential advantages but also possible disadvantages of this approach will be analyzed.
The diagnostic quality will also be researched. The assessment of the situation by the tele-EMS physician and the EMS physician on-scene will be matched to the definitive diagnoses of the in-hospital phase. A tracking of patients admitted to the hospitals and analysis of their data will be conducted, to compare the prehospital and in-hospital diagnoses. In connection with the evaluation of the diagnostic quality the length of hospital stay should also be taken into account. A prehospital diagnosis that is confirmed within the in-hospital phase probably supports the recovery of the patient and maybe the length of hospital stay is shortened in comparison with the control group. This evaluation is not the first line aim of the research project but the economic dimension of the project should not be negated. All personnel involved in the research project Med-on-@ix are aware of a sensible interpretation of economic data.
Archiving of and access to data related to the emergency mission (e.g., still pictures and audio communication data between EMS physician on-scene and tele-EMS physician) are conducted according to the principle of „parsimony of data and avoidance of data (minimal data storage)“ (according to § 3a BDSG = data security law of Germany). Video data of the video camera that is embedded into the ceiling of the ambulance is not recorded at all. Still pictures are saved – like mentioned above – specially protected together with the documentation of the mission. Audio data will be stored for seven days to allow short-term questions about the communication. After this time span audio data will be deleted automatically and a further protection from unauthorized access is not longer necessary. The short-term data storage is not conducted for reasons of patient treatment and has no influence on these processes. If still pictures should also be necessary for reasons of further scientific evaluation, the patient's consent must be obtained or the material will be technically edited, that it is anonymized. A provision of personal data to unauthorized third parties does not occur. Thus, the data remains in the circle of researchers involved in patient care.

The research project is designed for the testing of an improved system of emergency care. This includes the organizational and logistical optimization of the entire chain of survival. In order to avert risks and damages associated with the use of non validated methods for treatment of emergency patients, potential risks and benefits have to be weighted up against each other carefully, also to avoid a withholding of a potential

superior method. The project Med-on-@ix pursues the objective of a stepwise improvement of the emergency care. This target is a superior legal interest over the extremely narrow interpretation of data privacy. With the data storage and evaluation of data „as needed“ the ethical desire of a restricted use of private data is taken into account without preferring a group because of an anticipated benefit.

Finally, it should be emphasized again that in principle all standard treatment options are available, as in the regular emergency medical service also. The teleconsultation system is available as an additional system for evaluation purposes and every EMS physician has the right to decide to disregard this additional system.

Involved Personnel:

Physicians of the Department of Anesthesiology, University Hospital Aachen

Dr. med. Max Skorning (chief investigator)

Dr. med. Stefan Beckers

Dr. med. Sebastian Bergrath

Dr. med. Daniel Rörtgen

Dr. med. Jörg Brokmann (EMS chief)

Harold Fischermann

In-Sik Na

Univ.-Prof. Dr. Phil. Dipl.-Psych. Siegfried Gauggel (advisory function)

Heiko Schmitz M. A.

Responsible Facility:

Department of Anesthesiology, University Hospital Aachen
Head: Univ.- Prof. Dr. Rolf Rossaint
University Hospital Aachen
Pauwelsstraße 30
52074 Aachen
Germany

Dr. med. Max Skorning
